# GenIO: a phenotype-genotype analysis web server for clinical genomics of rare diseases

**DOI:** 10.1186/s12859-018-2027-3

**Published:** 2018-01-27

**Authors:** Daniel Koile, Marta Cordoba, Maximiliano de Sousa Serro, Marcelo Andres Kauffman, Patricio Yankilevich

**Affiliations:** 1 Instituto de Investigación en Biomedicina de Buenos Aires (IBioBA), CONICET - Partner Institute of the Max Planck Society, Buenos Aires, Argentina; 2 Consultorio de Neurogenética, Centro Universitario de Neurología y División Neurología, Hospital J.M. Ramos Mejia, Facultad de Medicina, UBA, Buenos Aires, Argentina; 3 Programa de Medicina de Precisión y Genómica, Instituto de Investigaciones en Medicina Traslacional, Facultad de Ciencias Biomédicas, Universidad Austral-CONICET, Buenos Aires, Argentina

**Keywords:** Rare disease, Exome sequencing, Genome sequencing, Clinical informatics, Variant analysis, Bioinformatics

## Abstract

**Background:**

GenIO is a novel web-server, designed to assist clinical genomics researchers and medical doctors in the diagnostic process of rare genetic diseases. The tool identifies the most probable variants causing a rare disease, using the genomic and clinical information provided by a medical practitioner. Variants identified in a whole-genome, whole-exome or target sequencing studies are annotated, classified and filtered by clinical significance. Candidate genes associated with the patient’s symptoms, suspected disease and complementary findings are identified to obtain a small manageable number of the most probable recessive and dominant candidate gene variants associated with the rare disease case. Additionally, following the American College of Medical Genetics and Genomics and the Association of Molecular Pathology (ACMG-AMP) guidelines and recommendations, all potentially pathogenic variants that might be contributing to disease and secondary findings are identified.

**Results:**

A retrospective study was performed on 40 patients with a diagnostic rate of 40%. All the known genes that were previously considered as disease causing were correctly identified in the final inherit model output lists. In previously undiagnosed cases, we had no additional yield.

**Conclusion:**

This unique, intuitive and user-friendly tool to assists medical doctors in the clinical genomics diagnostic process is openly available at https://bioinformatics.ibioba-mpsp-conicet.gov.ar/GenIO/.

**Electronic supplementary material:**

The online version of this article (10.1186/s12859-018-2027-3) contains supplementary material, which is available to authorized users.

## Background

The advances in genetics, and the growing availability of health and genetic data, are making personal genomics a clinical reality. Clinical implementation of whole-genome sequencing or whole-exome sequencing as a single and primary test, will provide a higher diagnostic yield than conventional testing, while decreasing the number of genetic tests and ultimately the time required to reach a genetic diagnosis [1]. Genetic risk communication and genetic diagnosis will rapidly broadened in scope and practice, as emerging genomic technologies allow more medical doctors to access information regarding their patients’ genetic makeup [2].

**Fig. 1:**
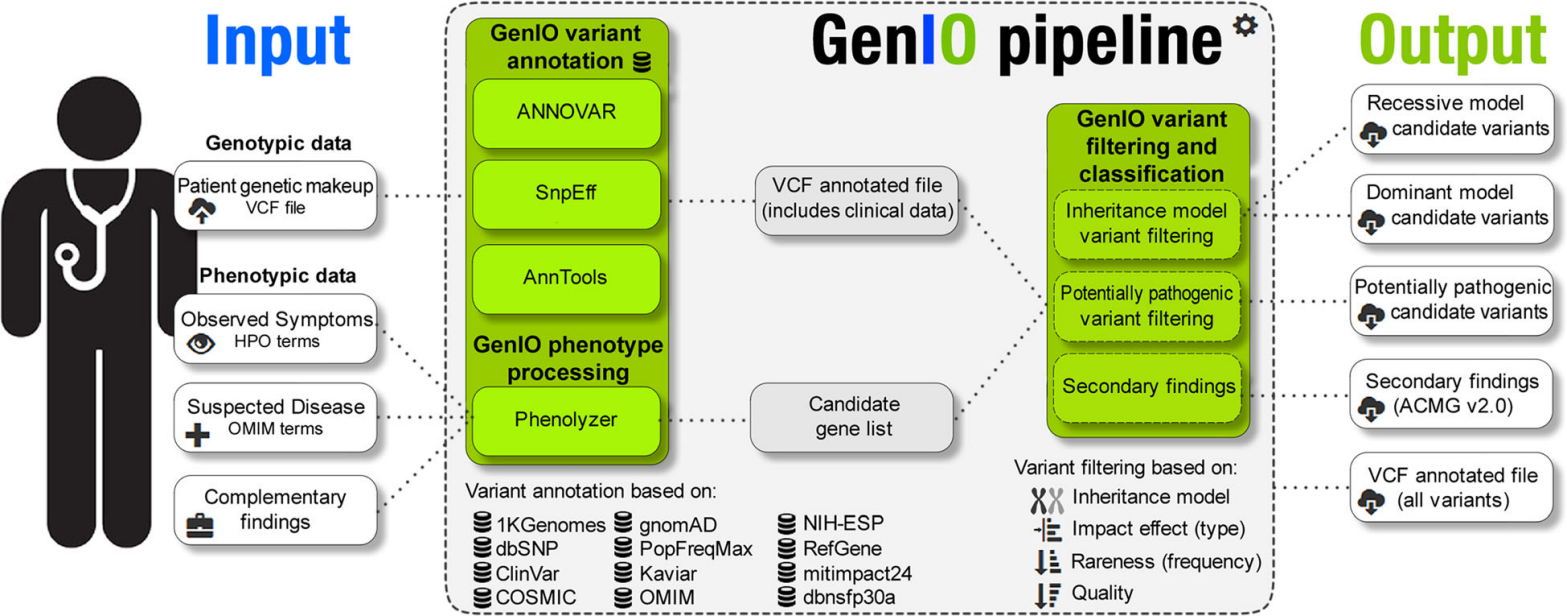
The GenIO pipeline. From left to right: Input parameters (VCF file and phenotype terms), GenIO pipeline, Output files (candidate recessive and dominant variants lists, potential pathogenic variants list, annotated VCF and secondary findings)

Here we present GenIO, a clinical genomics webtool to assist in the clinical genomics diagnostic process. Through our web server the user uploads the patient’s genetic information as a variant call format (VCF) file, and enter the patient’s clinical information as structured, comprehensive and well-defined terms for observed symptoms, suspected disease and complementary findings. Starting from thousands of variants, GenIO applies different annotations and filters, in order to identify a small number of the most probable recessive and dominant variants associated with rare Mendelian diseases (Fig. 1).

GenIO clinically classifies all variants using up-to-date clinical information, and identifies those variants with potentially functional pathogenic effects guided by the ClinVar database annotations [3], the Mendelian Clinically Applicable Pathogenicity (M-CAP) classifier [4], and the InterVar clinical interpretation [5] which follows the American College of Medical Genetics and Genomics and the Association for Molecular Pathology (ACMG-AMP) recommendations [6]. Additionally, GenIO reports secondary findings, in alignment with the ACMG latest recommendations for reporting of secondary findings in clinical exome and genome sequencing [7].

The GenIO process assists the medical practitioner in confirming a diagnosis for the patient case. At the moment, this crucial and time consuming annotation and filtering procedure is being done either manually, by the few geneticists able to benefit from bioinformatical support, or by using more complex web servers such as wAnnovar [8], Omim Explorer [9], eXtasy [10], PhenIX [11] or Phen-Gen [12], designed for research exploration and not for medical doctors working the diagnosis of rare diseases.

**Fig. 2:**
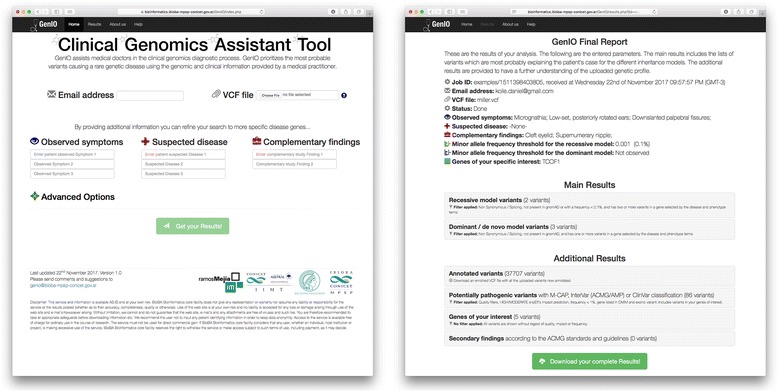
The GenIO user interface

GenIO interface has been designed to minimize usage complexity, allowing medical doctors to input a patient’s genetic makeup from a VCF file together with the patient’s phenotype, entered as controlled vocabulary terms from the Human Phenotype Ontology (HPO) project [13] and the Online Mendelian Inheritance in Man (OMIM) database (https://omim.org/) in a precise and easy way, to obtain a clear and concise output report (Fig. 2). This simple, intuitive and user-friendly clinical genomics Input-Output process gives GenIO its name.

GenIO is a unique web server, designed for medical doctors and researchers in the field of clinical genomics who may not have the necessary bioinformatics skills to annotate, classify and filter variants identified in high-throughput-sequencing studies to be able to choose the candidate disease causative gene from a small number of the most probable pathogenic variants associated with rare Mendelian disorders.

## Implementation

### Benchmark datasets

A benchmark dataset were simulated using a trustable and freely available source of pathogenic variants in the ClinVar public archive, their HPO associated terms, and the exome of a healthy individual to create a set of 125 simulated cases to be tested with GenIO. The ClinVar archive version 20,160,302 was downloaded and processed to filter out non-pathogenic variants, variants with no solid support evidence, and variants that lacked an OMIM registry. The resulting pathogenic gene variants were annotated with their known HPO associated terms downloaded from the Human Phenotype Ontology’s project website. Then a publicly available VCF file of the exome of a healthy individual was obtained [14]. Finally, 125 pathogenic gene variants with HPO annotations were randomly chosen from the filtered and annotated ClinVar file, and each added to different copies of a the exome of a healthy individual, obtaining a dataset of 125 simulated cases (pairs of VCF and HPO terms) to be tested in GenIO. The benchmark dataset is available at https://bioinformatics.ibioba-mpsp-conicet.gov.ar/GenIO/tests.zip

An additional real dataset of 40 patients from the Neurogenetics Unit in Hospital Ramos Mejía, Buenos Aires, which were previously studied applying WES and Sanger confirmation with a diagnostic rate of 40% (16 from 40) [15] were used to conduct a retrospective study. The study was approved by the Ethics Committee and Institutional Review Board of our Hospital JM Ramos Mejia, informed written consent was obtained from the participants, and the data were analyzed anonymously.

Finally, to further evaluate and compare the performance of GenIO, a smaller benchmark dataset of 10 cases with definitive diagnosis obtained from the former datasets was created to challenge other existing clinical genomics web servers to find the causative gene under the same input parameters.

### VCF file validation

The uploaded VCF file is validated in order to check compliance with the standardized VCF format version 4.0 or higher. The VCF header should contain the format information, and the column names and order as specified by the Global Alliance for Genomics and Health Data Working group file format team (https://samtools.github.io/hts-specs/). VCF columns must be tab separated, have each the proper data type, and have no duplicated variant entries. Only variants that have passed all the quality controls, and hence have a PASS value in the FILTER field, will be taken in consideration for the analysis. In this first release the files uploaded to GenIO must be 200 MB or smaller.

### Variant annotation and phenotype processing

GenIO’s variant annotation process uses Annovar [16], Anntools [17], and SnpEff [18] to annotate all variants with information from some of the main clinical genomics databases such as ClinVar, OMIM, the Genome Aggregation Database (gnomAD) [19], and dbSNP [20]; generating a merged and annotated VCF file.

GenIO’s phenotype process analyses the symptoms, suspected disease and complementary findings entered terms with Phenolyzer [21] to obtain the list of genes related to the patient’s disease/phenotype. Since the candidate genes associated to the entered phenotype are obtained by using Phenolyzer, which has an algorithm to predict putative disease genes, GenIO is then able to identify disease mutations in genes not previously described as being disease-causing.

### Variant filtering and classification

GenIO’s variant filtering and classification process identifies the most probable recessive and dominant deleterious variants in the list of genes, related to the patient’s disease/phenotype, by filtering on variant effect, population frequency, potential impact, and quality by using the variant_reduction script from Annovar and several custom filters. The default inheritance model output lists include deleterious variants with gnomAD Exome allele frequencies < 0.1% for the recessive model, and not observed in gnomAD for the dominant model. These variants are then classified by the Mendelian Clinically Applicable Pathogenicity (M-CAP) classifier, the InterVar ACMG-AMP clinical interpretation tool, and the ClinVar clinical significance annotation for the medical doctor to have a better understanding of the candidate causative variants informed. The GenIO’s interface advanced options enables the user to enter a specific gene list of interest for analysis, and to modify the filtering thresholds of population frequency according to the rareness of the suspected condition due to default filtering frequencies might be too low for several Mendelian disorders.

An additional list of variants with potentially functional disease-related pathogenic effects is generated by filtering variants in genes involved in Mendelian disorders (present in the OMIM database); with impact on the gene product (nonsense and frameshift mutations, splice site alterations, loss of stop codons, non-synonymous substitutions and codon insertions and deletions); with gnomAD Exome allele frequency < 1%; and with a clinical significance of pathogenic or likely-pathogenic nature, obtained either from the ClinVar database, the M-CAP classifier, or the InterVar ACMG-AMP clinical interpretation.

GenIO creates a minimum list of secondary findings, which includes deleterious variants found in 59 medically actionable genes (ACMG SF v2.0), recommended for reporting in clinical genomic sequencing studies.

### Server security

The GenIO application runs on a Secure HTTP Apache web server hosted on our Bioinformatics core facility at the Instituto de Investigación en Biomedicina de Buenos Aires (IBioBA). All GenIO databases and third-party programs used are locally installed on the server, so there is no further information transferred. The user data uploaded in the server is used for GenIO analysis only, stored for one month, and erased afterwards.

### Implementation and availability of web server

The methodology for identifying the most probable variants causing a rare disease described above is implemented in the web server named GenIO, using Linux, Apache, PHP, JavaScript architecture and is made publicly available online at https://bioinformatics.ibioba-mpsp-conicet.gov.ar/GenIO/

## Results

In order to validate the tool, we conducted a retrospective study on 40 patients with a diagnostic rate of 40% (16 from 40 cases) from the Neurogenetics Unit in Hospital Ramos Mejía, Buenos Aires. We reanalysed them with GenIO, obtaining, in the final inherit model output lists, all the known genes that were previously considered as disease causing (Additional file 1: Table S1). In previously undiagnosed cases, we had no additional yield. GenIO was also successfully validated with different well known cases such as Miller syndrome in Ng et al., 2010 [22], Nature Genetics (causative gene: *DHODH*), and with Schinzel-Giedion syndrome in Hoischen et al., 2010 [23], Nature Genetics (causative gene: *SETBP1*), both included as examples in the GenIO web server.

The benchmarking performed on GenIO with the simulated dataset identified the candidate pathogenic gene variants in the recessive or dominant inheritance models in 94 out of the 125 cases, obtaining a sensitivity of the 75.2%. It should be noted that the inheritance model filters applied in GenIO (see Implementation section) do not rely on the ClinVar clinical significance annotations, making this benchmark completely unbiased. All these tests were run with GenIO default parameters.

We compared GenIO with other existing clinical genomics webtools in terms of features and usability from a clinician user perspective. The compared web servers are wAnnovar, Omim Explorer, eXtasy, PhenIX and Phen-Gen (Table 1).

**Table 1:** Comparison with other web servers features

Feature \ Web servers	PhenIX	eXtasy	OMIM Explorer	Phen-Gen	wAnnovar	GenIO
Year of last update	2014	2013	2016	2013	2015	2017
Simple Input interface	Yes	Yes	No	Yes	Yes	Yes
Acceptance of VCF files	Yes	Yes	Yes	Yes	Yes	Yes
Phenotype (HPO terms)	Yes	Yes	Yes	IDs only	Yes	Yes
Phenotype (OMIM terms)	No	No	Yes	No	Yes	Yes
List of genes of interest to analyze	No	No	Yes	No	No	Yes
Customizable Rareness of the condition	Yes	No	Yes	Yes	Yes	Yes
Fast responsive interface	Yes	Yes	No	Yes	Yes	Yes
No need of extra files to run	Yes	Yes	Yes	No	Yes	Yes
Trio datasets	No	No	No	Yes	No	No
Freeform text input	No	No	Yes	No	No	No
Email notification	No	Yes	No	Yes	Yes	Yes
Dominant and Recessive in single run	Yes	No	Yes	No	Yes	Yes
Can be used by a medical practitioner	Yes	No	Yes	No	Yes	Yes
Output interface with results	Yes	No	Yes	No	Yes	Yes
Simple Output interface	Yes	–	No	–	Yes	Yes
ACMG-AMP Classification	No	No	No	No	No	Yes
M-CAP Classification	No	No	No	No	No	Yes
ClinVar Classification	Top 20 only	No	No	No	Yes	Yes
ACMG Secondary findings	No	No	No	No	No	Yes
Full VCF annotation	No	No	No	No	Yes	Yes
Handful set of candidate variants	Yes	No	Yes	Yes	Yes	Yes
Session saving	No	Yes	Yes	Yes	Yes	Yes
Phenotype suggestion	No	No	Yes	No	No	No
Secure server (https)	No	No	No	No	No	Yes
Multi-nucleotide variants	Yes	No	Yes	Yes	Yes	Yes

Finally, to further evaluate the performance of GenIO, we evaluated these same web servers on clinical results comparing 10 of the former analysed cases with definitive diagnosis to find the causative gene under the same input parameters (Additional file 1: Table S2).

## Discussion

GenIO results may enable diagnosis confirmation, and the output information will eventually help to identify the optimal treatment and clinical management for the patient. If, after analysis, the patient still lacks a clear etiology, the output information from GenIO can be used to launch a query on Matchmaker Exchange [24] platform to find additional cases with a deleterious variant in the same listed genes or with overlapping phenotype, which may provide sufficient evidence to identify the causative gene.

The quality of the variants identified in the VCF file uploaded by the user represents limitations to this clinical genomics analysis system. Since the raw sequences or genotype data is pre-processed and filtered before it is saved in a VCF format file, we are not able to ensure the quality of previous data processing, and have to assume an acceptable variant quality, and therefore a trustworthy variant call. We do, nevertheless, validate the format of the VCF file and filter out variants that did not pass the quality thresholds.

Although trio analysis is necessary for the detection of de novo mutations, GenIO does not support this analysis. As the list of de novo variants is usually small enough to be manually interpretable, usually does not require further interpretation.

The manual update of the GenIO’s annotation databases represents another limitation to the predictive performance. While clinical research evidence is being generated at ever faster rates, much of this evidence is not readily available in databases. Quality of the databases is also a possible limitation, as clinical databases may include wrong annotations. GenIO works with trustable sources, but nevertheless, they still could contain errors.

## Conclusions

GenIO’s intuitive and user-friendly interface was designed to be used not only by clinical genomics researchers, but also by medical doctors. Its simple input interface and the use of controlled vocabulary to enter clinical information minimize spelling and writing errors while entering the patient’s phenotypic information. Its diagnosis-oriented output presents only a small manageable number of the most probable recessive and dominant candidate gene variants associated with the rare disease case. Most of the existing clinical genomics web servers supporting diagnosis tasks are scientifically oriented and not designed to be used by medical doctors, on which we experienced some usability problems. In this sense, GenIO is one of the first public web servers developed with the aim of bringing new clinical genomics tools to the medical and scientific community.

Future work will include the identification of pharmacogenomic variants, the development of integrative visualizations for an improvement in the variant clinical interpretation, migration to a cloud computing architecture to handle bigger datasets, the development of a natural language processing of electronic medical records for phenotype suggestions, and the implementation of more ACMG-AMP guidelines and standards.

## Availability and requirements

Project name: GenIO.

Project home page:https://bioinformatics.ibioba-mpsp-conicet.gov.ar/GenIO/

Operating system(s): Platform independent.

Programming language: Javascript, PHP, GNU-bash shell.

Other requirements: Phenolyzer (v.1.0.5), Annovar (v.2017Jul17)(v.2015Dec14), Anntools (v.1.1), and SnpEff (v.4.2).

License: GNU General Public License.

Any restrictions to use by non-academics: licence needed.

## Additional file

Additional file 1: Table S1. Validated exomes with definitive diagnosis. **Table S2.** GenIO performance comparison. (DOCX 77 kb)

## Abbreviations

ACMG SF: ACMG Secondary Findings
ACMG-AMP: American College of Medical Genetics and Genomics and the Association of Molecular Pathology
gnomAD: The Genome Aggregation Database
HPO: Human Phenotype Ontology
IBioBA: Instituto de Investigación en Biomedicina de Buenos Aires
M-CAP: Mendelian Clinically Applicable Pathogenicity
OMIM: Online Mendelian Inheritance in Man
VCF: Variant Call Format
