# Supplementary material for: GenIO: a phenotype-genotype analysis web server for clinical genomics of rare diseases
Source: BMC Bioinformatics. 2018 Jan 27;19:25. doi: 10.1186/s12859-018-2027-3 (PMC5787240; doi:10.1186/s12859-018-2027-3)
Supplement: Additional file 1: Table S1. — Validated exomes with definitive diagnosis. Table S2. GenIO performance comparison. (DOCX 77 kb) [file 12859_2018_2027_MOESM1_ESM.docx]

Additional Information for

**GenIO: a phenotype-genotype analysis web server for clinical genomics of rare diseases**

Daniel Koile^1§^, Marta Cordoba^2,3§^, Maximiliano de Sousa Serro^1^, Marcelo Kauffman^2,3*^, Patricio Yankilevich^1*^

^1^ Instituto de Investigación en Biomedicina de Buenos Aires (IBioBA) - CONICET - Partner Institute of the Max Planck Society, Buenos Aires, Argentina

^2^ Consultorio de Neurogenética, Centro Universitario de Neurología y División Neurología. Hospital J.M. Ramos Mejia, Facultad de Medicina, UBA, Buenos Aires, Argentina

^3^ Programa de Medicina de Precisión y Genómica, Instituto de Investigaciones en Medicina Traslacional, Facultad de Ciencias Biomédicas, Universidad Austral-CONICET, Buenos Aires, Argentina

**Table S1. Validated exomes with definitive diagnosis**. In a retrospective study, all the causative genes of these cases were identified in the GenIO inherit model lists.

| CASE ID | CAUSATIVE GENE | PHENOTYPE | DEFINITIVE DIAGNOSIS OMIM | FAMILY HISTORY/VARIANT SEGREGATION | MUTATION(S) | TYPE OF MUTATION |
| --- | --- | --- | --- | --- | --- | --- |
| 1 | GRIK2 | Mental retardation, autism, epilepsy, dystonia | 611092 | Recessive  (both parents inheritance) | NM_021956.4:c592C>T; p.Arg198ter  Homozygous | nonsense |
| 2 | DEPDC5 | Epilepsy with Variable Foci | 604364 | Dominant  (paternal inheritance) | NM_001242896:c.4718T>C;p.Leu1573Pro | missense |
| 4 | CACNA1A | Hemiplegic Migraine | 141500 | Sporadic  (De novo) | NM_000068: c.3675C>A; p.Phe1225Leu | missense |
| 5 | STUB1 | Sporadic ataxia | 607207 | Sporadic  (both parents inheritance) | NM_005861.2:c.612+1 G> C; p.?  NM_005861.2:c.823C>G;Leu275Val | splicing /missense |
| 6 | SPG11 | Paraparesis, mental retardation, thinning of the corpus callosum peripheral neuropathy | 604360 | Sporadic  (both parents inheritance) | NM_025137:c.6763insA;p.Gln2242His  NM_025137:6726A>T;p.Leu2255HisfsX83 | frameshift  missense |
| 8 | KCNA2 | Ataxia, absence epilepsy, neurodevelopmental delay | 616366 | Sporadic  (*de novo*) | NM_001204269::c.G890A:p.Arg297Glu # | missense |
| 9 | DMD | Myopathy with very mild muscle weakness, hyperCKemia | 300377 | Sporadic | NM_004006.2:c.1149+1C>A | splicing |
| 11 | APTX | Ataxia, chorea, tremor, oculomotor apraxia | 208920 | Recessive  (both parents inheritance) | NM_175069.1:c.879G>A; p.Trp293ter #  Homozygous | nonsense |
| 21 | PCDH19 | Epileptic encephalopathy with partial seizures and ataxia | 300088 | Sporadic  (paternal inheritance) | NM_001184880:exon1:c.T1151G:p.Val384Gly | nonsense |
| 22 | PEX12 | Neonatal adrenoleukodystroph y with hepatic dysfunction, hypotonia, white matter lesions on MRI | 266510 | Sporadic  (both parents inheritance) | NM_000286:c.733_734insGCCT:p.Leu245fs #  NM_000286:c.533_535del:p.178_179del | frameshift  nonframeshift |
| 26 | POLR3A | Leukodystrophy with ataxia, cognitive impairment, abnormal ocular movements and symmetric hypo myelination on MRI | 607694 | Recessive  (both parents inheritance) | NM_007055.3:c.3781G>A; p.Glu1261Lys  NM_007055.3:c.3014G>A;p.Arg1005His # | missense  missense |
| 28 | SGCG | Myopathy with proximal muscular weakness, muscular atrophy | 608896 | Sporadic | NM_000231:exon6:c.521delT:p.Phe175LeufsX20 | frameshift |
| 29 | GNAO1 | Glycosylation congenital disorder with microcephaly, seizures, muscular weakness | 615473 | Sporadic  (*de novo*) | NM_020988:c.709G>A:p.Glu237Lys | missense |
| 32 | ALS2 | Spastic Quadriplegia, pyramidal dysfunction, fasciculation, muscular atrophy | 607225 | Sporadic | NM_020919:exon13:c.T2531A:p.Leu844His | missense |
| 39 | ATP7B | Sub acute Dementia with movement Disorders | 277900 | Recessive  (both parents inheritance) | NM_000053:c.2165T>A:p.Leu722Gln NM_000053:c.3704G>A:p.Gly235Asp | missense  missense |
| 40 | GNAO1 | Dyskinesia, Chorea, Hemiballismus | 615473 | Sporadic  (*de novo*) | NM_020988:c.709G>A:p.Glu237Lys | missense |

**Table S2. GenIO performance comparison.**

| Case ID (# variants - Causative Gene) | PhenIX | eXtasy^1^ | OMIM Explorer | Phen-Gen^2^ | wAnnovar^2^ | GenIO |
| --- | --- | --- | --- | --- | --- | --- |
| Miller (37,298 - DHODH) | Yes | - | Yes | Yes | Yes | **Yes** |
| Schinzel-Giedion (37,299 - SETBP1) | Yes | - | No | Yes | No | **Yes** |
| 1 (174,380 - GRIK2) | No** | - | No* | Yes | No | **Yes** |
| 5 (296,693 - STUB1) | No** | - | No* | No | No | **Yes** |
| 6 (167,544 - SPG11) | Yes | - | No* | Yes | Yes | **Yes** |
| 28 (168,206 - SGCG) | No | - | No* | No | No | **Yes** |
| 29 (183,996 - GNAO1) | No | - | No* | No | No | **Yes** |
| 40 (92,692 - GNAO1) | Yes | - | No** | No | No | **Yes** |
| 0 (37,297 - DVL1) | No | - | No | No | Yes | **Yes** |
| 368 (37,297 – CC2D2A) | No | - | No | No | Yes | **Yes** |

^1^ Causative gene not clearly denoted.

^2^ Causative gene among the top genes.

* Size error: There are too many variants present in uploaded VCF file.

** Other errors.
